# Use of the Endophytic Fungus *Daldinia cf*. *concentrica* and Its Volatiles as Bio-Control Agents

**DOI:** 10.1371/journal.pone.0168242

**Published:** 2016-12-15

**Authors:** Orna Liarzi, Einat Bar, Efraim Lewinsohn, David Ezra

**Affiliations:** 1 Department of Plant Pathology and Weed Research, Agricultural Research Organization, the Volcani Center, Rishon LeZion, Israel; 2 Newe Ya'ar Regional Research Center, Ramat Yishai, Israel; James Hutton Institute, UNITED KINGDOM

## Abstract

Endophytic fungi are organisms that spend most of their life cycle within plant tissues without causing any visible damage to the host plant. Many endophytes were found to secrete specialized metabolites and/or emit volatile organic compounds (VOCs), which may be biologically active and assist fungal survival inside the plant as well as benefit their hosts. We report on the isolation and characterization of a VOCs-emitting endophytic fungus, isolated from an olive tree (*Olea europaea* L.) growing in Israel; the isolate was identified as *Daldinia cf*. *concentrica*. We found that the emitted VOCs were active against various fungi from diverse phyla. Results from postharvest experiments demonstrated that *D*. *cf*. *concentrica* prevented development of molds on organic dried fruits, and eliminated *Aspergillus niger* infection in peanuts. Gas chromatography–mass spectrometry analysis of the volatiles led to identification of 27 VOCs. On the basis of these VOCs we prepared two mixtures that displayed a broad spectrum of antifungal activity. In postharvest experiments these mixtures prevented development of molds on wheat grains, and fully eliminated *A*. *niger* infection in peanuts. In light of these findings, we suggest use of *D*. *cf*. *concentrica* and/or its volatiles as an alternative approach to controlling phytopathogenic fungi in the food industry and in agriculture.

## Introduction

Endophytes are microorganisms that spend part of their life cycle within plant tissues without causing any visible damage or eliciting any defense reaction in host plants [1,2]. Endophytes benefit their hosts in various and variable aspects of fitness, such as growth enhancement, and tolerance to biotic and abiotic stresses [3]. Many endophytes isolated from trees were found to secrete specialized metabolites and complex glycoproteins [4–8] that might contribute to fungal survival in hostile environments [9], probably by improving an endophyte's ability to compete with other microorganisms for nutrients and space inside the plant tissues. They might simultaneously benefit the host by promoting its growth, protecting it from pathogens and pests, and increasing its survival under unfavorable conditions [2,10,11].

Some endophytic fungi can emit volatile organic compounds (VOCs) [12], which may be biologically active. A well-studied example of a VOC-emitting fungus is *Muscodor albus* [5], which first was isolated from a cinnamon tree in Honduras [5], and subsequently from other tree species in various parts of the world [13–16]. Other VOC-emitting fungi, such as *Ascocoryne* spp. [17], *Phoma* spp. [18], and the yeast-like *Aureobasidium pullulans* [19,20] were isolated and characterized for their volatiles profiles and bioactivity against postharvest and other plant pathogens.

Most volatile compounds emitted from fungi are carbon-based small molecules [21,22]. The VOCs emitting endophytes benefit their host in various aspects. For example: activity against plant pathogens [23], enhancement of host survival in desert habitats [18], inhibition of seed germination and thereby supporting the host in its competition with other plants [24, 25, 26], and involvement in repelling or attracting insects [21,27–34].

Examples of VOCs emitting biocontrol agents are *M*. *albus* or *A*. *pullulans*, which are used for postharvest control of plant pathogenic fungi [19,20,35,36], or insects [37]. Another possible application is the use of fungi that produce VOCs as a source of biofuel components [23,38,39]. Although the use of endophytes for biocontrol presents much promise [40–42] there are many challenges to be overcome, because of the complexity of the system–the endophyte/host associations are highly variable [43]. In the present paper we report on the isolation and characterization of an endophytic VOC-emitting fungus that was isolated from an olive tree (*Olea europaea* L.) growing in Israel. This isolate was found to be very similar to members of the well characterized genus, *Daldinia*; it was extensively reviewed by Stadler et al. [44]. Although *Daldinia* species are known to produce volatiles with characteristic fruity odors [45], in most studies the volatiles have not been identified [44,46,47]; to the best of our knowledge, only one study identified, analyzed and compared volatiles emitted by *D*. *hawksworthii*–a new species of *Daldinia*–with those emitted by *D*. *concentrica* [48]. Therefore, the objectives of the present study were to characterize and identify volatile compounds emitted by *D*. *cf*. *concentrica*, and to examine the antimicrobial activity of the fungus and its volatile compounds *in vitro*, within a search for possible future commercial applications.

## Materials and Methods

### Fungal isolation, maintenance and growth conditions

The *D*. *cf*. *concentrica* isolate that was cultured for use in the present study was obtained as an endophyte from a branch of an olive tree (*Olea europaea* L.) located in the Ha'Ela Valley in the Judean Hills in Israel (N 31.681915, E 34.988792). Wood fragments were surface-sterilized by immersion in ethanol for 10 s, followed by flaming. Then, small pieces were cut and placed on potato dextrose agar (PDA) (Acumedia, Lansing, Michigan, USA) amended with tetracycline at 12 μg/mL (Sigma, Rehovot, Israel), and incubated at 25°C. After 5 days, isolated fungal hyphal tips that emerged from the plant material onto the PDA were removed with a sterile scalpel and transferred to a new PDA-tetracycline plate. A single spore colony was used throughout this study. The culture was maintained routinely on PDA-tetracycline plates and incubated at 25°C. Fresh fungal mycelium was transferred to a new plate every 2 weeks. The fungus was stored for longer periods either by freezing small pieces of PDA harbouring mycelia of the fungus in 30% glycerol at –80°C or by growing the fungus on autoclaved sweet corn seeds at 25°C.

The *D*. *cf*. *concentrica* isolate was grown on various natural and commercial media. All the natural media–corn flour, crushed wheat, lentils, rice, corn, chickpea, and oats–were bought in commercial stores, soaked with water, and autoclaved. Of the commercial media: PDA, potato dextrose broth (PDB), nutrient agar (NA), Luria-Bertani (LB) agar, and tryptic soy agar were purchased from Acumedia (Lansing, Michigan, USA); lima bean agar was purchased from Difco (Detroit, Michigan, USA); and agar-agar for the agar-water medium was purchased from Romical (Be'er Sheva, Israel). All synthetic media were prepared according to their manufacturers' instructions.

Test fungi and oomycetes *Alternaria alternata* pathotype tangelo, *A*. *alternata*, *Aspergillus niger*, *Botrytis cinerea*, *Colletotrichum* sp., *Coniella* sp., *Fusarium euwallaceae*, *F*. *mangiferae*, *F*. *oxysporum*, *Lasiodiplodia theobromae*, *Neoscytalidium dimidiatum*, *Penicillium digitatum*, *Phoma tracheiphila*, *Pythium aphanidermatum*, *P*. *ultimum*, *Rhizoctonia solani*, and, *Sclerotinia sclerotiorum* (D. Ezra, lab collection) were grown on PDA amended with tetracycline at 12 μg/mL, and incubated at 25°C; except for *Pythium* sp., which was grown on PDA without tetracycline.

#### Isolation of fungal DNA

Half-square-centimeter squares were cut with a sterile scalpel from 7-day-old, single-spore mycelial cultures grown on PDA at 25°C. The agar was scraped from the bottom of each piece to exclude as much agar as possible from the isolation procedure. The pieces were homogenized in liquid nitrogen with a mortar and pestle, and DNA was extracted by means of the GenElute Plant Genomic DNA Miniprep Kit (Sigma, Rehovot, Israel) according to the manufacturer’s instructions.

#### Amplification of internal transcribed spacer 5.8S rDNA and partial actin gene

The internal transcribed spacer (ITS) region was amplified by using primers ITS1 (TCCGTAGGTGAACCTGCGG) and ITS4 (TCCTCCGCTTATTGATATGC) [49]. Part of the actin gene was amplified by using primers ACT512F (ATGTGCAAGGCCGGTTTCGC) and ACT783R (TACGAGTCCTTCTGGCCCAT) [50]. Amplifications were done in a 25-μL reaction mix containing 10–20 ng of DNA, 1 μL (10 μM) of each primer, dNTPs (2.5 mM each), 2.5 μL of reaction buffer, 0.125 μL (0.625 U) of DreamTaq DNA polymerase (Fermentas, Vilnius, Lithuania), and PCR-grade ddH_2_O (Thermo Fisher Scientific, Vilnius, Lithuania). Amplifications were performed in a Personal Cycler (Biometra, Göttingen, Germany).

The PCR program for ITS was as follows: denaturizing at 96°C for 5 min; followed by 35 cycles of 96°C for 45 s, 55°C for 45 s, and 72°C for 1 min; followed by 5 min at 72°C. The PCR program for actin was similar to the one for ITS, except that the denaturizing temperature was 95°C, and the number of cycles was 40. PCR products were examined by electrophoresis in a 1.2% agarose gel [51]. The PCR products of ITS and actin were purified by using the DNA Clean & Concentrator-5 purification kit (Zymo Research, Irvine, California, USA) according to the manufacturer’s instructions. Purified products were sent for direct PCR sequencing by Macrogen (Amsterdam, Netherlands).

Sequences of ITS and partial actin gene were submitted to GenBank and deposited as accession numbers EU201138 and FJ269018, respectively. The sequences obtained in the present study were compared with those already present in the GenBank database by applying the BLAST software on the National Center for Biotechnology Information website (http://www.ncbi.nlm.nih.gov/BLAST/).

#### *D*. *cf*. *concentrica* bioactivity tests

The activity of *D*. *cf*. *concentrica* volatiles was examined by means of the "Sandwich Method", which prevents any direct contact between *D*. *cf*. *concentrica* and the test fungus or oomycte. Thus, any effect of the former on growth of the latter should be due only to the volatiles produced by *D*. *cf*. *concentrica*, which spread freely across the plates. A plug of PDA harboring the *D*. *cf*. *concentrica* mycelia was added to a 50-mm Petri dish containing 5 mL of PDB, or whichever growth medium was to be examined, and allowed to grow for 3–4 days at 25°C. Then, a plug of PDA harboring mycelia of the test fungus or oomycete was placed in another 50-mm Petri dish containing PDA, and the dish with the test fungus or oomycete was put on top of the dish containing *D*. *cf*. *concentrica*. Both Petri dishes, without their covers, were connected with parafilm and their contents were allowed to grow at 25°C. The effect of *D*. *cf*. *concentrica* on the test fungus or oomycete was examined after 2 days, by comparing the growth of the test fungus or oomycete with that of the same fungus or oomycete in the absence of *D*. *cf*. *concentrica*. All experiments were performed in triplicate.

The inhibitory effect of *D*. *cf*. *concentrica* on various plant pathogenic fungi or oomycete was examined as described above, except that *D*. *cf*. *concentrica* was grown for 6 days prior to the addition of the test fungi or oomycete, and the inhibition was examined after 6 days of incubation. At the end of the assay, the viability of each test fungus or oomycete was evaluated by transferring inoculum plugs to fresh PDA plates and observing the growth that developed within the next 2 days.

The temperature range that supported *D*. *cf*. *concentrica* growth and activity was examined as follows: *D*. *cf*. *concentrica* was grown in 50-mm Petri dishes containing 5 mL of PDB at various temperatures – 10, 15, 18, 20, 22, and 25°C–and growth was monitored for 6 days. For activity tests at 15 and 18°C, *D*. *cf*. *concentrica* was grown for 7 days at these temperatures in 50-mm Petri dishes containing 5 mL of PDB, before addition of *A*. *niger* as the test fungus. The two fungi were connected in the "Sandwich Method" as described above, and the growth of the test fungus was assessed after 4 days, and compared with that of *A*. *niger* grown under the same conditions in the absence of *D*. *cf*. *concentrica*. The activity test at 10°C was performed in a similar manner, except that: the test fungi were *A*. *alternata*, *B*. *cinerea*, and *P*. *digitatum* instead of *A*. *niger*, which did not grow at 10°C, even in the absence of the volatiles; *D*. *cf*. *concentrica* was grown for about 1 month before introduction of the test fungi; and the test fungi were exposed to *D*. *cf*. *concentrica* volatiles for 13 days.

Organic dried plums, raisins, and apricots were bought commercially. The experiment was performed in triplicate, with two biological repetitions, in sealed 1-L boxes. Each box housed zero, one, or two 50-mm Petri dish(es) containing 5 mL of PDB and a plug of *D*. *cf*. *concentrica*. The fungi were allowed to grow for 3–4 days at 25°C, after which a 120-g sample of each dried fruit was incubated at room temperature for 3–4 h with excess sterile double-distilled water. The swollen dried fruit samples were then each placed in a 50-mm Petri dish and the dishes were placed in the boxes with *D*. *cf*. *concentrica* or in the control boxes without the fungus. The boxes were further incubated at 25°C for 6–9 days before fungal appearance on the swollen dried fruits was assessed.

Peanuts were bought commercially and prearranged in 50-mm Petri dishes in the presence of 5 mL of sterile double-distilled water. There were four peanuts per dish, with triplicated treatments, and two biological repetitions. Then, each of the peanuts was inoculated with three 10-μL drops of *A*. *niger* conidial suspension containing 10^6^ conidia/mL. Next, each Petri dish with peanuts was transferred to a sealed 1-L box that contained zero, one, or two 50-mm Petri dish(es) containing *D*. *cf*. *concentrica* that had been pre-grown for 3–5 days at 25°C. The boxes were further incubated for 10 days at 25°C before *A*. *niger* development on the peanuts was assessed.

#### Volatiles identification

*Daldinia cf*. *concentrica* was grown on 5 mL of PDB in 20-mL sealed solid-phase microextraction (SPME) vials. A plug of growing mycelium was placed in each vial and incubated at 25°C for 3 days. The vial was then preheated to 40°C for 15 min after which an automatic HS-SPME MPS2 syringe (Gerstel, Mülheim, Germany) with a 65-μm polydimethylsiloxane/divinylbenzene/carboxen (PDMS/DVB/CAR) fiber (Supelco, Bellefonte, PA, USA) was inserted into the sample headspace for 25 min. The exposed SPME syringe was then inserted into the injector port of a GC-MS apparatus for 10 min. Volatile compounds were analyzed on a 6890/5973N GC-MSD apparatus (Agilent Technologies, San Diego, CA, USA) equipped with an Rxi-5 SIL MS fused-silica capillary column that measured 30 m × 0.25 mm × 0.25 μm in length, diameter, and bore (Restek, Bellefonte, PA, USA). Helium at a constant pressure of 9.1 psi was the carrier gas. The injector temperature was 250°C, and splitless injection was used. The detector temperature was 280°C. The oven temperature was held at 50°C for 1 min, then increased to 180°C at a rate of 5°C/min, and then to 280°C at 25°C/min. The recorded mass range was 41 to 350 m/z, with electron energy of 70 eV. A mixture of straight-chain alkanes (C7-C23) was injected into the column under the above conditions, for determination of retention indices. The GC-MS spectrum profiles were analyzed with the ChemStation software (Agilent Technologies, Waldbronn, Germany). The volatiles were identified by comparison of their retention indices with published values and with spectral data obtained with standards or from the W9N08 and HPCH2205 GC-MS libraries, and NIST Mass Spectral Library, ver. 2.0d. Comparable analyses were applied to SPME vials containing only PDB, and the identified compounds were subtracted from those found in the vials containing the fungus.

For quantitative analysis, samples were prepared by mixing 13 g of sample and 5 g of NaCl with chlorobenzene, and the mixture was injected into the GC-MS. All samples were prepared in duplicate. For the chemical compounds – 3-methyl-1-butanol, (±)-2-methyl-1-butanol, 4-heptanone, isoamyl acetate, and *trans*-2-octenal–confirmatory identification was made by comparing the GC-MS data of fungal products with those of available authentic standards, obtained from Sigma (Rehovot, Israel).

### Chemical mixtures bioactivity tests

All chemical compounds were purchased from Sigma (Rehovot, Israel) and were of the highest purity available. The bioactivity of the mixtures was determined as follows. Petri dishes, 90 mm in diameter, with air volume of 80 mL, contained 15 mL of growth medium comprising PDA amended with tetracycline at 12 μg/mL. The dishes were inoculated, in triplicate, with two plugs of the each test fungi: *A*. *alternata* and *B*. *cinerea* in the same dish, and *P*. *digitatum* and *A*. *niger* in separate dishes. A disconnected cover from an Eppendorf tube was placed in the middle of the dish, to which was added a series of increasing volumes–ranging from 0 to 200 μL–of the mixture. The dishes were then sealed with parafilm and incubated at room temperature for 2 days, after which growth of the test fungi in those dishes was compared with that in mixture-free control dishes. The ability of two mixtures–designated as "Mixture A" and "Mixture B"–to control other plant pathogenic fungi or oomycete was determined as described above, except that the concentration of the mixture was constant at 1 mL/L and growth inhibition was estimated after 6 days. The viability of the test fungi or oomycete after exposure to the mixtures was examined as described for *D*. *cf*. *concentrica*.

The activity of each component of the mixture was examined separately, as described for the mixtures. For "Mixture A" the volumes of 3-methyl-1-butanol, (±)-2-methyl-1-butanol, 4-heptanone, and isoamyl acetate, were 16, 16, 32, and 16 μL, respectively. For "Mixture B" the volumes of 4-heptanone and *trans*-2-octenal were each 40 μL.

The ability of the mixtures to inhibit the growth of *A*. *alternata*, *B*. *cinerea*, *P*. *digitatum*, and *A*.*niger* was examined at temperatures of 4, 10, 15, 18, 20, 22, and 25°C. The experiment was performed in sealed 1-L boxes, with three boxes for each temperature. Each box contained four uncovered PDA Petri dishes, i.e., one for each test fungus, and the mixture was located on the opposite side of the box. "Mixture A", at 1 mL/L, was held in a (12 × 35)-mm vial (S Murray & Co, Surrey, England), whereas "Mixture B", at 0.05 mL/L, was placed on (8 × 3)-cm sheets of laboratory absorbent paper. For each temperature, one control box containing triplicates of each of the four test fungi, and no mixture, was prepared. The boxes were incubated for 2 weeks and then fungal growth was evaluated.

Wheat grains were bought commercially and prearranged in triplicated 50-mm Petri dishes, with 8 g of wheat grains per plate. There were two biological repetitions. Sealed 1-L boxes were loaded with one Petri dish with wheat grains, one Petri dish with 5 mL of distilled water, and an (8 × 3)-cm sheet of laboratory absorbent paper soaked with Mixture A or Mixture B at 0, 0.25, 0.5, or 1 mL/L. The boxes were incubated for 10 days at 25°C and then appearance of fungus on the wheat grains was evaluated visually.

The effects of the mixtures on *A*. *niger* development on peanuts were examined with the following setup, which was triplicated, with two biological repetitions. Petri dishes, each containing four peanuts in the presence of 5 mL of sterile double-distilled water were incubated in a sealed 1-L box, in the presence of a (12 × 35)-mm vial containing Mixture A at 1 mL/L, at room temperature. Half of the peanuts had been pre-inoculated with *A*. *niger* conidial suspension containing 10^6^ conidia/mL, as described above. Inoculated or control, uninoculated peanuts were incubated under the same conditions, in the absence of the mixture. Intrinsic and artificial development of *A*. *niger* was evaluated after 10 days. Mixture B was similarly examined, except that: the incubation time was 8 days; instead of a vial the mixture was soaked into an (8 × 3)-cm sheet of laboratory absorbent paper at concentrations of 0.0, 0.05, 0.25, and 0.5 mL/L; and the peanuts were not artificially inoculated with *A*. *niger*, but rather the fungus developed from an intrinsic source. The effects of individual chemical compounds on peanut germination and *A*. *niger* development were examined as follows. Four peanuts in each of two 50-mm Petri dishes were placed in sealed 1-L boxes in the presence of 5 mL of distilled water. The chemical compounds – 3-methyl-1-butanol, (±)-2-methyl-1-butanol, 4-heptanone, and isoamyl acetate–in concentrations of 0.0, 0.25, 0.5, 0.75, 1.0, and 1.25 mL/L, in separate (12 × 35)-mm vials were placed in each box, there being one vial per box. Half of the peanuts, i.e., the four peanuts in one Petri dish per box, were artificially inoculated with *A*. *niger* as described above. The boxes were incubated at room temperature for 1 week, after which peanut germination and *A*. *niger* establishment were evaluated. The effect of *trans*-2-octenal on peanut germination was examined similarly except that the concentration of the compound was 1 mL/L, the peanuts were not inoculated with *A*. *niger*, and the incubation time was 4 days.

## Results

### Fungal isolation and characterization

The fungal isolate used in this study was obtained as an endophyte from a small branch of an olive tree (*Olea europaea* L.) located in the Ha'Ela Valley in central Israel. Pure fungal colonies grown on PDA generated fast-growing whitish hyphae that reached the edge of the agar dish at 25°C within 6–8 days, after which the mycelium became woolly in appearance. The hyphae, which measured 1.2–2.0 μm in width, commonly grew by branching; no septa were observed. During growth the hyphae became green to gray in color, with brown to black spots that appeared first in older mycelia and later spread throughout the colony surface. The conidia began to appear 4 days after inoculation, and were dark green to black in color. The conidia continued to emerge from the mycelium during its growth, and appeared in clusters, usually oval in shape and measuring 1.6 × 2.4 μm; they branched from the sides or ends of the hyphae. In addition, after 3–4 days of growth, the fungus produced volatiles with a pronounced, sweet and fruity odor, which is a known feature of *Daldinia* species [44]. Thus, these characteristics suggest that this fungal isolate belongs to the genus *Daldinia*.

Molecular characterization of the fungal isolate, based on 100% coverage, revealed 100% identity of the sequences of the ITS 5.8S rDNA region and the partial actin gene–approximately 500 and 200 bp, respectively–with the corresponding sequences of *Daldinia concentrica* published as accession numbers AM292045 andKC551906, respectively, in GenBank. Partial sequences of β-tubulin and RNA polymerase II subunit 2 (M. Stadler, personal communication) confirmed the identification. Therefore, based on the morphological characteristics and molecular identification, we conclude that our isolate belongs to the *Daldinia concentrica* complex. Since a concise identification of the species in this complex is dependent on teleomorph availability, we refer our isolate as *D*. *cf*. *concentrica*.

#### Fungal bioactivity

The presence of the odor led to the hypothesis that the volatiles emitted by this fungus might have antimicrobial activity. We tested the capability of *D*. *cf*. *concentrica* to grow and emit bioactive VOCs on various plants as food sources and on commercial media such as corn flour, crushed wheat, lentils, rice, corn, chickpeas, oats, PDA, 0.25 PDA, PDB, NA, LB, tryptic soy agar, lima bean agar, and agar-water. We found that although the fungus was able to grow on all the tested media, its activity varied among them. For example, wheat, corn, and rice supported the capability of *D*. *cf*. *concentrica* to inhibit *A*. *niger* growth–by 85, 65, and 54%, respectively–but not as well as the commercial media PDA and PDB, both of which elicited 100% inhibition. However, the medium that supported the highest bioactivity of the fungal VOCs was PDB. This result is based on the finding that although *A*. *niger*, *Botrytis cinerea*, and *Penicillium digitatum* were fully inhibited in both PDA and PDB, 100% inhibition of *Alternaria alternata* occurred only in the latter medium, whereas in the former medium there was only 51% inhibition. Furthermore, the viability of the test fungi differed between the solid and the liquid media. Whereas all the four test fungi–*A*. *niger*, *B*. *cinerea*, *A*. *alternata*, and *P*. *digitatum*–that were exposed to *D*. *cf*. *concentrica* grown on PDB were killed, only *A*. *niger* and *P*. *digitatum* were dead after exposure to *D*. *cf*. *concentrica* grown on PDA. Therefore, we used PDB media throughout this study. Interestingly, *D*. *cf*. *concentrica* can be stored on dry corn grains at 25°C for at least 2 years without its viability being impaired.

We also examined the temperature range within which *D*. *cf*. *concentrica* was able to grow and emit biologically active VOCs. We found that the temperature ranges of fungal growth and of its biological activity overlapped between 10 and 25°C, at which it elicited full inhibition of *A*. *alternata*, *B*. *cinerea*, *A*. *niger*, and *P*. *digitatum*.

Among 17 plant-pathogenic fungi and oomycetes tested, growth of 12 fungi was fully inhibited (Table 1). However, in some cases, full inhibition was temporary, and the test fungi were still viable and started to grow after removal of *D*. *cf*. *concentrica* volatiles. As shown in Table 1, *D*. *cf*. *concentrica* inhibited the growth of pathogens from diverse phyla, such as Ascomycota, Basidiomycota, and Oomycota (Stramenopiles).

**Table 1:** Effects of the volatile compounds of *D*. *cf*. *concentrica* and artificial mixtures on tested plant pathogenic fungi and oomycete

	*D*. *cf*. concentrica*		Mixture A**		Mixture B	
Pathogen	Growth inhibition***	Viability****	Growth inhibition	Viability	Growth inhibition	Viability
***Alternaria alternata* pathotype tangelo**	100.0	-	100.0	-	100.0	-
***Alternaria alternata***	100.0	-	100.0	-	100.0	-
***Aspergillus niger***	100.0	-	98.0	+	100.0	-
***Botrytis cinerea***	100.0	+	100.0	-	100.0	-
***Colletotrichum* sp.**	100.0	+	100.0	+	100.0	-
***Coniella* sp.**	100.0	+	100.0	+	100.0	-
***Fusarium euwallaceae***	81.60	+	96.96	+	100.0	-
***Fusarium mangiferae***	100.0	+	100.0	+	100.0	-
***Fusarium oxysporum***	69.60	+	95.92	+	100.0	-
***Lasiodiplodia theobromae***	0.0	+	95.0	+	100.0	-
***Neoscytalidium dimidiatum***	100.0	-	94.0	+	100.0	-
***Penicillium digitatum***	100.0	-	95.0	+	100.0	-
***Phoma tracheiphila***	100.0	-	100.0	-	100.0	-
***Pythium aphanidermatum***	9.60	+	93.33	-	96.67	-
***Pythium ultimum***	30.80	+	96.67	-	96.67	-
***Rhizoctonia solani***	100.0	-	98.0	+	100.0	-
***Sclerotinia sclerotiorum***	100.0	-	100.0	-	100.0	-

* *D*. *cf*. *concentrica* was grown for 6 days on PDB prior to its exposure to test fungi or oomycete.

** The concentration of the mixtures was 1 mL/L air space.

*** Growth inhibition after 6 days was calculated as percentage inhibition compared with that of a control grown under the same conditions in the absence of *D*. *cf*. *concentrica* or mixtures.

**** Viability of the tested fungi or oomycete after 6 days of exposure to *D*. *cf*. *concentrica* or mixtures.

**Fig 1:**
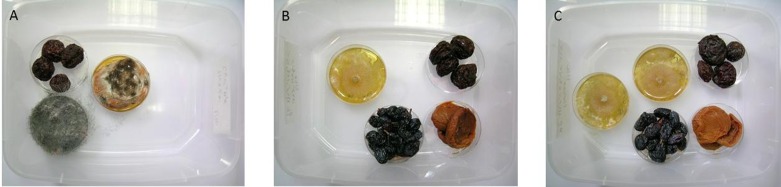
Prevention of fungal damage by *D*. *cf*. *concentrica* volatiles on organic dried fruits. (A) Control swollen fruits. (B) Swollen fruits in the presence of one culture dish of *D*. *cf*. *concentrica*. (C) Swollen fruits in the presence of two culture dishes of *D*. *cf*. *concentrica*.

Exposure of organic dried fruits to *D*. *cf*. *concentrica* volatiles resulted in full disinfection of the fruits relative to the controls (Fig 1). Swelling of the fruit in water induced the appearance of plant pathogenic fungi such as *Rhizopus* sp., *Penicillium* sp., and *Aspergillus* sp. (Fig 1A). In contrast, the presence of one (Fig 1B), or two (Fig 1C) *D*. *cf*. *concentrica* culture dishes abolished the appearance of all pathogenic fungi.

**Fig 2:**
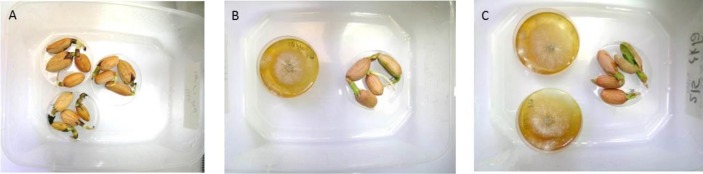
Disinfecting effect of *D*. *cf*. *concentrica* volatiles on peanuts. (A) *A*. *niger* inoculated peanuts. (B) *A*. *niger* inoculated peanuts in the presence of one culture dish of *D*. *cf*. *concentrica*. (C) *A*. *niger* inoculated peanuts in the presence of two culture dishes of *D*. *cf*. *concentrica*.

Similarly, the disinfecting activity of *D*. *cf*. *concentrica* also was shown in peanuts (Fig 2). However, in this experiment the peanuts were artificially inoculated with *A*. *niger*. The *D*. *cf*. *concentrica* VOCs fully prevented *A*. *niger* growth on the peanuts without affecting their germination.

#### Chemical composition of the volatiles

In order to further understand the basis of the bio-activity of *D*. *cf*. *concentrica* VOCs, we chemically analyzed the gas phase of the fungus grown on PDB with a GC/MS apparatus. As shown in Table 2, we tentatively identified 27 different compounds that could be divided among several classes of chemical substances: alcohols, dienes, ketones, aldehydes, and sesquiterpenes. Eight compounds–methyl-1,4-cyclohexadiene, phenyl ethyl alcohol, 3-methyl-1-butanol, (±)-2-methyl-1-butanol, 4-heptanone, 3-methoxy-2-naphthol, isoamyl acetate, and *trans*-2-octenal–suggested by the GC/MS analysis, were commercially available. It should be noted that although the fungus emitted 2-octenal of unknown stereochemistry, in our experiments we used only *trans*-2-octenal, because only this isomer was commercially available. We purchased these compounds and examined their ability to control the growth of *A*. *niger*, *B*. *cinerea*, *A*. *alternata*, and *P*. *digitatum*; we found that only phenyl ethyl alcohol and 3-methoxy-2-naphthol failed to inhibit fungal growth. One compound–methyl-1,4-cyclohexadiene–exhibited poor growth inhibition of *A*. *niger* and *P*. *digitatum*–by 10.8 and 3.1%, respectively–and therefore was not further included in this study. Final identification of the five remaining compounds – 3-methyl-1-butanol, (±)-2-methyl-1-butanol, 4-heptanone, isoamyl acetate, and *trans*-2-octenal–was based on comparison with authentic standards. The standards yielded retention times and mass spectra that were identical to those of the fungal products only for the first three compounds; the last two compounds have been only tentatively identified on the basis of database comparisons. The abundances of the validated compounds were 5.9, 2.4, and 0.08 ppm for 3-methyl 1-butanol, (±)-2-methyl 1-butanol, and 4-heptanone, respectively. It is interesting to note that in contrast to *Muscodor albus*–another VOC-emitting endophytic fungus–the possibly carcinogenic naphthalene [25] was not identified among the *D*. *cf*. *concentrica* VOCs.

**Table 2:** Compounds emitted by *D*. *cf*. *concentrica*

Retention time (min)	Suggested compound*	Molecular formula	Main fragments (m/z)	Area (%)	MW
**2.2**	3-methyl-1-butanol	C_5_H_12_O	42, **55**, 57, 70	1.0	88
**2.25**	2-methyl-1-butanol	C_5_H_12_O	41, 56, **57**, 70	1.1	88
**2.7**	1-methyl-1,3-cyclohexadiene	C_7_H_10_	77, **79**, 91, 94	6.5	94
**2.8**	1-methyl-1,4-cyclohexadiene	C_7_H_10_	77, **79**, 91, 94	3.9	94
**4.1**	4-heptanone	C_7_H_14_O	**43**, **71**, 114	0.2	114
**4.3**	isoamyl acetate	C_7_H_14_O_2_		traces	130
**5.2**	4-heptyn-2-ol	C_7_H_12_O	45, 53, 67 **68**, 97, 112	4.2	112
**5.5**	2-octenal	C_8_H_14_O	42, 55, **57** 84, 98, 126	33.3	126
**6.3**	octanal	C_8_H_16_O	69, 71, **72** 83, 84, 95 110, 128	4.7	128
**7.2**	4,4-dimethyl-1,3-cyclopentanedione	C_7_H_10_O_2_	41, **56**, 126	4.0	126
**7.7**	2,2,5-trimethylcyclopentanone	C_8_H_14_O	41, **56**, **126**	16.5	126
**10.5**	phenyl ethyl alcohol	C_8_H_10_O	65, **91**, 92, 122	1.9	122
**18.2**	β-elemene	C_15_H_24_	41, 53, 55, 67, 68, 79, 81, **93**, 107, 121, 133, 135, 147, 149, 161, 189, 204	0.06	204
**18.4**	β-elemene	C_15_H_24_	41, 53, 55, 67, 68, 79, 81, **93**, 107, 121, 133, 135, 147, 149, 161, 189, 204	1.0	204
**19.4**	(+)-α-funebrene	C_15_H_24_	-	traces	-
**19.6**	α-guaiene	C_15_H_24_	41, 53, 55, 67, 79, 81, 93, **105**, 121, 133, 147, 161, 189, 204	0.1	204
**19.9**	2-(4-hydroxyphenyl)ethanol	C_8_H_10_O_2_	?	0.08	204
**20.7**	terpenes	C_10_H_10_O_3_	108, **136**, 137, 163, **178**	9.0	178
**21.0**	β-selinene	C_15_H_24_	41, 53, 55, 67, 79, 81, 91, 93, **107**, 121, 133, 147, 161, 175, 189, 204	0.7	204
**21.2**	α-selinene	C_15_H_24_	41, 53, 55, 67, 79, 81, 91, 93, 107, 121, 133, 147, 161, 175, **189**, 204	0.2	204
**21.4**	α-bulnesene	C_15_H_24_	41, 53, 55, 67, 79, 81, 91, 93, **107**, 121, 133, 147, 161, 189, 204	0.7	204
**21.6**	germacrene A	C_15_H_24_	-	traces	-
**21.8**	7-epi-α-selinene	C_15_H_24_	-	traces	-
**22.0**	dauca-4(11),8-diene	C_15_H_24_	-	traces	-
**22.9**	veratryl acetone	C_11_H_14_O_3_	-	traces	-
**25.1**	3-methoxy-2-naphthol	C_11_H_10_O_2_	77, 131, 159, 174	1.6	174
**25.2**	pogostol	C_15_ H_26_O	41, 53, 55, 71, 81, 93, **107**, 121, 131, 147, 161, 189, 204	1.2	222

* identified according to NIST Mass Spectral Library, ver. 2.0 d

#### Biological activity of chemical mixtures

In order to chemically mimic the bioactivity of *D*. *cf*. *concentrica* against plant pathogenic test fungi, we prepared various mixtures, each containing two to four of the most active volatile compounds – 3-methyl-1-butanol, (±)-2-methyl-1-butanol, 4-heptanone, isoamyl acetate, and *trans*-2-octenal–in various ratios. Each mixture was tested against *A*. *niger*, *B*. *cinerea*, *A*. *alternata*, and *P*. *digitatum*. Two mixtures achieved the best results, i.e., at least 95% inhibition of these test fungi by the lowest concentrations of mixture. The mixtures were: "Mixture A", which contained 3-methyl-1-butanol, (±)-2-methyl-1-butanol, 4-heptanone, and isoamyl acetate in the proportions of 1:1:2:1; and "Mixture B", which contained equal amounts of 4-heptanone and *trans*-2-octenal. The ability of the mixtures to control 17 plant pathogenic fungi and oomycetes is presented in Table 1. These results demonstrate that Mixture B was more effective than Mixture A or *D*. *cf*. *concentrica*, in that it killed all the test fungi; furthermore, in most cases Mixture A was more effective than *D*. *cf*. *concentrica*, except against *Rhizoctonia solani*, *P*. *digitatum*, *Neoscytalidium dimidiatum*, and *A*. *niger*, all of which survived exposure to Mixture A but not to *D*. *cf*. *concentrica* volatiles. In addition, our results demonstrate that the activity of the mixtures, similarly to that of *D*. *cf*. *concentrica*, affected pathogens belonging to various phyla: Ascomycota, Basidiomycota, and Oomycota.

To elucidate whether the mixtures exhibited additive or synergistic effects with respect to each of their chemical constituents, we determined the growth inhibition and survival of *A*. *niger*, *B*. *cinerea*, *A*. *alternata*, and *P*. *digitatum* after exposure to the amount of each individual component contained in the mixture. As shown in Table 3, the additive or synergistic behavior of Mixture A depended on the pathogenic fungus tested: for *A*. *niger*, *B*. *cinerea*, and *A*. *alternata* Mixture A showed additive effects: each of the four components of the mixture contributed some level of inhibition. In contrast, however, Mixture A behaved synergistically toward *P*. *digitatum*: (±)-2-methyl-1-butanol and isoamyl acetate elicited low levels of inhibition – 18.7 and 7.3%, respectively–whereas 3-methyl-1-butanol and 4-heptanone failed to control fungal growth. Another difference between Mixture A and its chemical constituents was that whereas the mixture fully inhibited and killed *B*. *cinerea* and *A*. *alternata*, each of its components elicited only partial inhibition and allowed fungal survival. As shown in Table 4, *trans*-2-octenal was the main contributor to the effect of Mixture B; the effect of this compound was identical to that of the mixture (Table 1). Nevertheless, in light of our findings that the second component of Mixture B – 4-heptanone–played a role in biological control applications other than inhibiting and killing pathogenic fungi–it was effective against nematodes and aphids [52] (Ezra D. unpublished data)–we continued the experiments with Mixture B and not only *trans*-2-octenal.

**Table 3:** Biological activity of each chemical component consisting 1 mL/L (air space) of Mixture A

	3-methyl-1-butanol*		(±)-2-methyl-1-butanol*		4-heptanone**		isoamyl acetate*	
	Growth inhibition***	Viability****	Growth inhibition	Viability	Growth inhibition	Viability	Growth inhibition	Viability
***Aspergillus niger***	23.8	+	17.5	+	42.0	+	18.5	+
***Alternaria alternata***	28.4	+	40.5	+	64.2	+	38.4	+
***Botrytis cinerea***	45.7	+	39.1	+	79.5	+	39.1	+
***Penicillium digitatum***	0.0	+	18.7	+	0.0	+	7.3	+

* The concentrations was 0.2 mL/L air space.

** The concentrations was 0.4 mL/L air space.

*** Growth inhibition after 6 days was calculated as percentage inhibition compared with that of a control grown under the same conditions in the absence of the chemical compound.

**** Viability of the tested fungi after 6 days of exposure to the chemical compound

**Table 4:** Biological activity of each chemical component consisting 1 mL/L (air space) of Mixture B

	4-heptanone*		*trans*-2-octenal*	
	Growth inhibition**	Viability***	Growth inhibition	Viability
***Aspergillus niger***	6.8	+	100	-
***Alternaria alternata***	40.7	+	100	-
***Botrytis cinerea***	70.3	+	100	-
***Penicillium digitatum***	0	+	100	-

* The concentrations was 0.5 mL/L air space.

** Growth inhibition after 6 days was calculated as percentage inhibition compared with that of a control grown under the same conditions in the absence of the chemical compound.

*** Viability of the tested fungi after 6 days of exposure to the chemical compound.

Examination of the temperature range within which each of the mixtures was active revealed 75–100% inhibition of *A*. *niger*, *B*. *cinerea*, *A*. *alternata*, and *P*. *digitatum* by Mixture A, and 100% inhibition of these by Mixture B at temperatures in the range of 4–25°C. This result indicates the possibility of biotechnological use of the mixtures at low temperatures–at which *D*. *cf*. *concentrica* is unable to grow.

**Fig 3:**
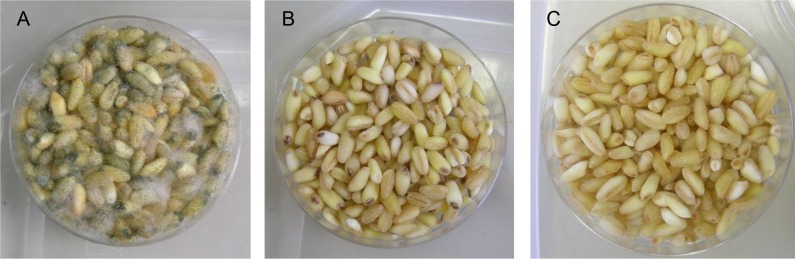
Disinfecting effect of chemical mixtures on commercial wheat grains. (A) Untreated wheat grains. (B) Wheat grains after exposure to Mixture A at 0.25 mL/L. (C) Wheat grains after exposure to Mixture B at 0.25 mL/L.

Possible applications of the mixtures as disinfectants were examined, with regard to storage of grains. Exposure of commercial wheat grains to Mixture A and Mixture B resulted in effective disinfection of the grains compared to the control (Fig 3).

**Fig 4:**
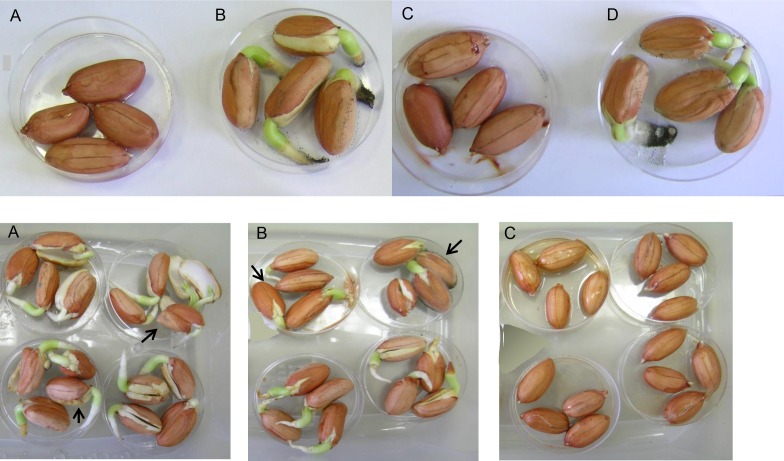
Disinfecting effect of chemical mixtures on peanuts. Upper panel–Mixture A: (A) Peanuts inoculated with *A*. *niger* in the presence of mixture at 1 mL/L. (B) Peanuts inoculated with *A*. *niger* in the absence of mixture. (C) Uninoculated peanuts in the presence of mixture at 1 mL/L. (D) Uninoculated peanuts in the absence of mixture. Lower panel–Mixture B: (A) Uninoculated peanuts in the absence of mixture. (B) Uninoculated peanuts in the presence of mixture at 0.05 mL/L. (C) Uninoculated peanuts in the presence of mixture at 0.5 mL/L. Arrows indicate the development of intrinsic *Aspergillus* sp.

Mixture A protected peanuts from development of both intrinsic and artificially inoculated *A*. *niger* (Fig 4, upper panel). However, in contrast to results obtained with *D*. *cf*. *concentrica* (Fig 2), exposure of peanuts to Mixture A resulted in loss of their ability to germinate. We found that among the chemical components of Mixture A, 3-methyl-1-butanol and isoamyl acetate prevented peanut germination, whereas exposure to (±)-2-methyl-1-butanol and 4-heptanone did not impair germination. Furthermore, none of these compounds fully inhibited *A*. *niger* inoculation (data not shown). Another chemical compound–*trans*-2-octenal–which is one of the components of Mixture B–permitted peanut germination. In light of the finding that both components of Mixture B permitted germination, we examined the ability of Mixture B to protect peanuts from *A*. *niger* infection without limiting their germination ability. As shown in Fig 4 (lower panel), peanut germination occurred in untreated peanuts as well as in those that had been exposed to low concentrations of Mixture B; however, high concentrations of Mixture B did inhibit peanut germination. Interestingly, prevention of intrinsic *A*. *niger* development occurred only under exposure to high concentrations of Mixture B, whereas at low concentrations, i.e., those that allowed peanut germination, *A*. *niger* could be clearly detected. Taken together, these results suggest that the use of our mixtures to protect peanuts from *A*. *niger* development should be recommended only in applications in which peanut germination is not needed.

## Discussion

The VOCs from endophytic *D*. *cf*. *concentrica* were found to exhibit antimicrobial activity against a wide range of fungi and oomycetes from diverse phyla. These biologically active VOCs also protected dried fruits, peanuts, and wheat grains from fungal attack, by either intrinsic or artificially inoculated fungi, which indicates potential for biotechnological use of the fungus and/or its VOCs. The use of endophytes as sources of bioactive products is widely known [21,40,41]. Examples include: endophytes producing antibiotics [42], endophytes used in the flavor and fragrance industry, and potential production of mycodiesel from volatile-producing endophytes [21,23,39,53,54]. Recently, reviews on bioactive microbial volatiles and their potential exploitation to improve plant growth, development, and health in a sustainable agricultural context were published by Kanchiswamy and colleagues [55,56].

Our present findings revealed differences in the bioactivity of *D*. *cf*. *concentrica* according to whether it was grown on solid or liquid forms of potato dextrose media. The higher activity obtained by growth on the liquid medium is not clear; however, in light of the findings that VOCs emitted by *Daldinia* spp. were dependent on the culture medium [48], and that production of VOCs by an endophytic fungus was affected by epigenetics [54], we can assume that even the minor shift from solid to liquid potato dextrose media was sufficient to influence the GC-MS profile of the VOCs and, therefore, their ability to control the growth of the test fungi. However, in the present study we did not compare the GC-MS profiles of the volatiles emitted by *D*. *cf*. *concentrica* grown on solid versus liquid medium, but we previously demonstrated the effect of substrate on the bioactivity of volatile antimicrobials produced by *M*. *albus* [57].

Our results show differences between the bioactivity of *D*. *cf*. *concentrica* and that of artificial mixtures of its volatiles. In most cases, the mixtures exhibited higher activity against plant pathogenic fungi and oomycetes, and a wider temperature range, than the intact fungus. This higher activity might be because there were higher concentrations of the chemical components in the synthetic mixtures than in the VOCs emitted by the fungus, and/or because of absence of other volatiles that could interfere with the disinfecting activity. Another observed difference was that exposure to the artificial mixtures elicited an herbicidal effect on peanuts (Fig 4), whereas the presence of *D*. *cf*. *concentrica* resulted in full disinfection of peanuts without affecting their germination (Fig 2). These results suggest that volatiles that were not included in the synthetic mixture might play a role in preservation of germinability. Conversely, it could be because there were higher concentrations of certain compounds in the mixtures than in the natural emissions. Generally, the possibility of using live microorganism for biocontrol faces several limitations; the scope of biological agents is limited by their need for food resources and suitable temperature and humidity conditions to enable them to be active and effective. Alternatively, using those microorganisms as new sources of active compounds might provide new, eco-friendly metabolites that exhibit properties equivalent to or even better than those of the live agent, without the limitations imposed by the need for life-supporting conditions.

One of the most disturbing problems associated with storage of seeds and foods is spoilage of products by various fungi. Moreover, some of these fungi secrete toxins into their surroundings–substances that might be harmful to human health: aflatoxins and fumonisin are examples of mycotoxins secreted by certain species of *Aspergillus* and *Fusarium*, respectively, which are potent carcinogens [58,59]. Attempts to control these pathogens involve chemical pesticides that are known to be harmful to livestock and humans [60]. Therefore, in light of our present results, we propose an alternative means to achieve this control by using safer compounds originating from a fungus. These may provide a basis for new "green control" products in food industries and in agriculture.

At least one-third of the compounds emitted by *D*. *cf*. *concentrica* were classified as sesquiterpenes. This is in accordance with the finding that terpenoids and polyketides were the most common anti-microbial secondary metabolites from endophytes [61], and the finding that *D*. *concentrica* produced sesquiterpenes [62]. Our tested compounds, the components of mixtures A and B, are known to exhibit antimicrobial activities. For example: it was previously shown that the compounds 3-methyl-1-butanol and 2-methyl-1-butanol produced by *Saccharomyces cerevisiae* exhibited strong antimicrobial activity against *Sclerotinia sclerotiorum* [63]. Also, 3-methyl-1-butanol was characterized as a cyanobacteriolytic agent [64], and growth inhibitor of the pathogen *Aspergillus flavus* [65]. A common volatile constituent of human urine is 4-heptanone [66,67], which also can be detected in bacteria such as *Collimonas* sp. [68], and *Burkholderia ambifaria* [69]. It was demonstrated that 4-heptanone exhibited antibiotic properties against *Clostridium botulinum* [70]. Isoamyl acetate, which emits a marked banana aroma and is one of the main components of Ginjo-Flavor, showed strong antifungal activity against various filamentous fungi [71]; it also showed antibacterial activity against *Escherichia coli*, in which it damaged cell membranes and altered protein expression [72]. Although *trans*-2-octenal, one of the main VOCs emitted by truffles [73], was found to be inactive against 11 bacterial pathogens of humans [74], it was shown to reduce aflatoxin production in corn, cottonseed, and peanuts [75], and to elicit phytotoxic effects on *Arabidopsis thaliana* [76] and neurotoxic effects on *Drosophila melanogaster* [34].

It should be noted that since most of the fungal VOCs in this study were tentatively identified using GC-MS followed by comparison to NIST database, we cannot rule out the possibility that the fungus produces additional metabolites, such as small polyketides–a known feature of *Daldinia* [77–79], which are too polar to be detected by the GC-MS, and/or are absent from the database as standards. Furthermore, it was previously demonstrated that unknown metabolites could be assigned to known VOCs on tentative identification using NIST database [48]. Thus, in order to gain the complete diversity of the fungal VOCs, further experiments involving total synthesis and/or preparative GC followed by NMR are needed.

Interestingly, all the compounds tested in the present study are used in the food industry **(**http://www.sigmaaldrich.com/industries/flavors-and-fragrances.html). Thus, although the mixtures have not yet been tested for toxicity against mammals, it is likely that it will be feasible to use them for preservation and microbial control in food. Furthermore, we consider that other *D*. *cf*. *concentrica* volatiles, which were not included in the mixtures we tested, may exhibit additional biological activities and therefore should be examined in the future.
